# HIV-1 Subtype C-Infected Individuals Maintaining High Viral Load as Potential Targets for the “Test-and-Treat” Approach to Reduce HIV Transmission

**DOI:** 10.1371/journal.pone.0010148

**Published:** 2010-04-12

**Authors:** Vladimir Novitsky, Rui Wang, Hermann Bussmann, Shahin Lockman, Marianna Baum, Roger Shapiro, Ibou Thior, Carolyn Wester, C. William Wester, Anthony Ogwu, Aida Asmelash, Rosemary Musonda, Adriana Campa, Sikhulile Moyo, Erik van Widenfelt, Madisa Mine, Claire Moffat, Mompati Mmalane, Joseph Makhema, Richard Marlink, Peter Gilbert, George R. Seage, Victor DeGruttola, M. Essex

**Affiliations:** 1 Harvard School of Public Health AIDS Initiative, Department of Immunology and Infectious Diseases, Harvard School of Public Health, Boston, Massachusetts, United States of America; 2 Botswana–Harvard AIDS Institute Partnership, Gaborone, Botswana; 3 Department of Biostatistics, Harvard School of Public Health, Boston, Massachusetts, United States of America; 4 Department of Dietetics and Nutrition, Robert R. Stempel School of Public Health, Florida International University, Miami, Florida, United States of America; 5 Department of Biostatistics, University of Washington, and Fred Hutchinson Cancer Research Center, Seattle, Washington, United States of America; 6 Department of Epidemiology, Harvard School of Public Health, Boston, Massachusetts, United States of America; 7 Division of Infectious Diseases, Vanderbilt University School of Medicine, Vanderbilt Institute of Global Health (VIGH), Nashville, Tennessee, United States of America; University of Cape Town, South Africa

## Abstract

The first aim of the study is to assess the distribution of HIV-1 RNA levels in subtype C infection. Among 4,348 drug-naïve HIV-positive individuals participating in clinical studies in Botswana, the median baseline plasma HIV-1 RNA levels differed between the general population cohorts (4.1–4.2 log_10_) and cART-initiating cohorts (5.1–5.3 log_10_) by about one log_10_. The proportion of individuals with high (≥50,000 (4.7 log_10_) copies/ml) HIV-1 RNA levels ranged from 24%–28% in the general HIV-positive population cohorts to 65%–83% in cART-initiating cohorts. The second aim is to estimate the proportion of individuals who maintain high HIV-1 RNA levels for an extended time and the duration of this period. For this analysis, we estimate the proportion of individuals who could be identified by repeated 6- vs. 12-month-interval HIV testing, as well as the potential reduction of HIV transmission time that can be achieved by testing and ARV treating. Longitudinal analysis of 42 seroconverters revealed that 33% (95% CI: 20%–50%) of individuals maintain high HIV-1 RNA levels for at least 180 days post seroconversion (p/s) and the median duration of high viral load period was 350 (269; 428) days p/s. We found that it would be possible to identify all HIV-infected individuals with viral load ≥50,000 (4.7 log_10_) copies/ml using repeated six-month-interval HIV testing. Assuming individuals with high viral load initiate cART after being identified, the period of high transmissibility due to high viral load can potentially be reduced by 77% (95% CI: 71%–82%). Therefore, if HIV-infected individuals maintaining high levels of plasma HIV-1 RNA for extended period of time contribute disproportionally to HIV transmission, a modified “test-and-treat” strategy targeting such individuals by repeated HIV testing (followed by initiation of cART) might be a useful public health strategy for mitigating the HIV epidemic in some communities.

## Introduction

HIV-infected individuals with high plasma viral load progress to AIDS faster [1], [2], [3], and are more likely to transmit virus [4], [5], than those with a lower viral load. As a modified version of the “test-and-treat” strategy [6], identification and antiretroviral (ARV) treatment of individuals who maintain high HIV-1 RNA levels for an extended period of time might represent an important public health strategy to significantly curtail HIV incidence.

An extensive body of literature supports the idea that higher levels of plasma viral load in HIV-1 infection are associated with higher transmission of HIV [4], [5], [7], [8], [9]. Each 0.5 log_10_ increment in HIV-1 RNA level may lead to a 40% greater risk of heterosexual transmission [10]. Studies focusing on mother-to-child-transmission (MTCT) demonstrate that levels of plasma viral RNA load [11], [12], [13] in HIV-infected mothers are the best predictors of viral transmission. Individuals with primary or late-stage HIV infection are highly infectious [14], [15] due to increased levels of viral RNA load. Although the individual benefits of starting combined ARV therapy (cART) in acute seroconverters remain uncertai, early initiation of cART may offer the secondary public health benefit of reducing transmission caused by those with recent seroconversion and higher viral loads.

Viral load dynamics following HIV-1 subtype B infection have been well characterized by previous studies [1], [16], [17], [18], [19], [20], [21]. The initial peak of viral load resolves in a steady-state viral set-point within four to six months. Individuals with higher viral set-points in HIV infection generally lose CD4+ cells more quickly, progress to AIDS more rapidly, and experience mortality sooner than those with lower HIV-1 RNA set-points. Mellors et al. demonstrated that 80% of individuals with viral load ≥30,000 (4.48 log_10_) copies/ml progress to AIDS within 6 years [1]. In the MACS cohort, the upper quartile of HIV-infected individuals maintained viral RNA load from 59,987 to 72,651 (4.78 to 4.86 log_10_) copies/ml for approximately 6 to 18 months post-infection [22], and those who progressed to AIDS within 3 years maintained levels of viral load over 4.5 log_10_ (Figure 2A in [22]). Recent HIV-1 subtype B-based studies from the USA and Canada [23], and the mainland USA and Hawaii [24] reported median viral RNA from 3.88 to 4.80 log_10_ with inter-quartile ranges from 2.7 to 4.9 log_10_ among the total number of 9,115 drug-naïve participants.

Limited data regarding levels and distribution of plasma viral RNA load are available for HIV-1 non-subtype B settings, and particularly for HIV-1 subtype C. Gray et. al reported median viral load in a cohort of 51 HIV-1 subtype C-infected individuals from Zambia, Malawi, Zimbabwe, and South Africa within the 3.82 to 4.02 log_10_ range during 2 to 24 months post-seroconversion [25]. In a cohort of 958 HIV-infected women attending antenatal clinics in Zambia the median viral RNA load was between 4.56 and 4.62 log_10_ [26]. The median viral load in a cohort of 62 acutely and recently HIV-1 subtype C-infected individuals from Botswana was 4.10 log_10_ [27]. The median (IQR) plasma HIV-1 RNA set point was estimated at 4.45 log_10_ (4.32 to 5.14 log_10_) in a cohort of 31 seroconverters from Malawi [28]. Median (IQR) plasma HIV-1 RNA in a cohort of 377 subtype C-infected infants from South Africa was as high as 5.90 (5.6–5.9) log_10_ [29], which is consistent with infants exhibiting higher levels of viral load than adults.

Utilizing data from clinical studies in Botswana, this study aimed to assess the levels and distribution of plasma viral RNA in HIV-1 subtype C infection, to identify the proportion of subjects who maintain high viral load for an extended period of time, and to determine how long such individuals sustain high viremia. The main rationale for employing data from cohorts representing different stages of HIV infection was to determine levels and distribution of plasma HIV-1 RNA in the local epidemic, and assess the HIV-1 RNA variability among different populations. While the clinically meaningful threshold of viral load affecting HIV transmission is unknown and is likely to be a continuum between 10,000 copies/ml and 100,000 copies/ml, we used 50,000 copies/ml as the threshold supported by the Quinn et al. [5] study that demonstrated that the highest HIV-1 transmission rates were in persons having plasma HIV-1 RNA levels greater than 50,000 (4.7 log_10_) copies/ml. We also estimated the proportion of individuals with high viral load that can be identified by repeated HIV testing (6-month- versus 12-month-interval testing) and the potential reduction of the period of high HIV transmissibility that can be achieved by repeated HIV testing and initiation of ARV treatment in the community.

## Methods

### Ethics statement

This study was conducted according to the principles expressed in the Declaration of Helsinki. The study was approved by the Institutional Review Boards of Botswana and the Harvard School of Public Health. All patients provided written informed consent for the collection of samples and subsequent analysis.

### Study participants and cohorts

Description of the Botswana–Harvard Partnership (BHP) studies has been presented elsewhere [30]. For the purposes of this study, baseline data were used from the following seven BHP cohorts that were monitored including extensive clinical and laboratory follow up for prolonged periods. The time of enrollment to each cohort is shown in Supplementary Table S1. Three types of cohorts were distinguished: general population, MTCT, and cART-initiating cohorts.

MTCT cohort BHP004, *Mashi study*: Prevention of milk-borne transmission of HIV-1C in Botswana (completed). The main goals of this project were two-fold. First, to assess whether the addition of a single dose of maternal nevirapine (NVP) at labor along with zidovudine (AZT or ZDV) from week 34 of gestation provides additional benefit in reducing HIV transmission from mother to child. The study was amended to determine whether maternal NVP (per HIVNET 012 protocol) is necessary in the setting of maternal ZDV from 34 weeks gestation through delivery and single-dose prophylactic infant NVP (at birth) plus ZDV (from birth to 4 weeks) for the reduction of HIV transmission from mother to child. The second goal was to determine the effectiveness and safety of prophylactic AZT to breast-feeding infants to prevent milk-borne HIV transmission. The baseline HIV RNA load in plasma was available for 1,189 Mashi participants. Results of the Mashi study were presented elsewhere [11], [31], [32], [33], [34].

cART-initiating cohort BHP007, *Tshepo study*: The adult antiretroviral treatment and drug resistance study (completed). The study was an open-label, randomized combination ARV study with a multi-factorial, 3x2x2 design. The factors included a comparison of three NRTI combinations (ZDV/lamivudine (3TC), ZDV/didanosine (ddI), and 3TC/stavudine (d4T)), a comparison of two NNRTIs (NVP and efavirenz (EFV)), and a comparison between two adherence strategies (standard of care (SOC) versus an intensified adherence strategy, SOC plus community-based supervision). The baseline HIV RNA load in plasma was available for 631 Tshepo participants. Results of the Tshepo study were presented elsewhere [35], [36], [37].

General population cohort BHP010, *Botsogo study*: A natural history of HIV-1 subtype C disease progression study (completed). This observational study gathered data on HIV-1 subtype C disease progression from ARV-naïve HIV-infected individuals with CD4+ cell count ≥400/mm^3^. The objectives of the study were (i) to determine the kinetics of HIV-1 subtype C disease progression (ii) to estimate the rate of CD4+ cell decline, and (iii) to analyze the time to first HIV-associated or AIDS-defining condition or death in persons with initial CD4+ cell count ≥400/mm^3^. The baseline HIV RNA load in plasma was available for 444 Botsogo participants.

General population cohort BHP011, *Dikotlana study*: Micronutrient therapy and HIV in Botswana (completed). The study was a randomized, multifactorial, double-blind placebo-controlled trial to determine the efficacy of micronutrient supplementation in improving immune function and preventing early mortality in HIV-1-infected adults whose CD4+ were >350 cells/mm^3^. The design compared the efficacy of multivitamins, or selenium, or the combination of multivitamins and selenium to a placebo supplementation. The baseline HIV RNA load in plasma was available for 842 Dikotlana participants.

MTCT cohort BHP016, *Mma Bana study*: A randomized trial of ZDV + 3TC + lopinavir/ritonavir vs. ZDV + 3TC + abacavir for virologic efficacy and the prevention of MTCT among breastfeeding women having CD4+>200 cells/mm^3^ in Botswana (ongoing). This study involved cART initiation by week 28 of gestation in breastfeeding women having CD4+>200 cells/mm^3^. The third group included pregnant women who received ZDV + 3TC (given as co-formulated Combivir™ or Lamzid™) + NVP as the National Program regimen because they had CD4+<200 cells/mm^3^. This group also breast-fed their infants. The baseline HIV RNA load in plasma was available for 726 Mma Bana participants.

cART-initiating cohort BHP019, *Mashi Plus study*: The study was designed to determine the response to NVP-containing cART among women who have previously taken single-dose NVP for the prevention of MTCT (completed). The baseline HIV RNA load in plasma was available for 302 Mashi Plus participants. Results of the study were reported elsewhere [32], [38].

cART-initiating cohort BHP026, *Bomolemo study*: A prospective cohort study evaluating the efficacy and tolerability of tenofovir and emtricitabine (given as co-formulated Truvada™) as the NRTI backbone for first-line cART in treatment-naïve adults (ongoing). The baseline HIV RNA load in plasma was available for 214 Bomolemo participants.

Although HIV-1 subtyping was not performed systematically for all individuals included in the seven BHP cohorts analyzed, our previous studies provide strong evidence for the overwhelming dominance of HIV-1 subtype C as the etiologic agent of the HIV/AIDS epidemic in Botswana [27], [39], [40], [41]. According to the HIV Sequence Database at LANL [42], 99.4% of the deposited 1,425 sequences from Botswana belong to HIV-1 subtype C. Therefore, we assume that the vast majority of subjects in this study are infected with HIV-1 subtype C.

Both baseline and longitudinal data were used from the eights cohort, BHP012 *Tshedimoso study*, n = 42, Markers of Viral Set Point in Primary HIV-1C Infection (ongoing). The study was designed to evaluate potential trends between viral load and viral genetic diversity in acute and early HIV-1 subtype C infection, to determine the relationship between virologic parameters and viral set-point, and to identify immunological parameters that correlate with viral set-point in primary HIV-1 subtype C infection. All subjects included in the longitudinal analysis were genotyped and were found to be infected with HIV-1 subtype C. Results of the study were reported elsewhere [27], [39], [41], [43], [44], [45]. The primary infection cohort was comprised of individuals with estimated time of seroconversion. For acutely infected subjects (n = 8) the time of seroconversion was estimated as the midpoint between the last seronegative test and the first seropositive test (within a week in most cases). For recently infected subjects (n = 34) the time of seroconversion was estimated by Fiebig stage assignment as described elsewhere [43], [45]. For a time zero we used the estimated time of seroconversion rather than the estimated time of HIV infection because frequent sampling in this study allowed reliable measurement of the time of seroconversion based on a series of laboratory tests, which can be more accurate than estimation of the time of HIV infection. Time points of sampling and HIV-1 RNA testing in the primary infection study (n = 42) are presented in the Supplementary Figure S1. Individuals whose CD4+ cell count dropped below 200 cells per cubic millimeter or developed opportunistic infection had access to antiretroviral therapy (Combivir (ZDV/3TC) 300/150 mg twice a day plus nevirapine 200 mg twice a day if female, or efavirenz 600 mg every day if male) free of charge, in accordance with Botswana National Treatment Program guidelines.

### Viral load testing

Plasma HIV-1 RNA was quantified by the COBAS Ampli-Prep/COBAS AMPLICOR HIV-1 Monitor Test, version 1.5, according to the manufacturer's instructions as described previously [27]. The method of viral load quantification used in the study has been certified by the Virology Quality Assurance at Rush University, Chicago, IL, as a part of the laboratory proficiency testing. The level of detection was from 50 (1.7 log_10_) copies/ml for the ultrasensitive method and 400 (2.6 log_10_) copies/ml for the standard method to 750,000 (5.88 log_10_) copies/ml. Analysis of individuals with estimated time of seroconversion from the Tshedimoso study included both pre- and post-cART data, which is clearly indicated in the presenting materials.

### Statistics

Descriptive statistics (mean and accompanying 95% confidence intervals, median and corresponding inter-quartile range) were quantified using Sigma Stat v. 3.5. Comparisons of continuous outcomes between two groups were based on the Mann-Whitney Rank Sum test. A Spearman rank correlation was used for analysis of potential associations between continuous variables. The Kolmogorov-Smirnov test was used to test whether the distribution of a continuous outcome follows a normal distribution. For the purpose of analysis in this study we defined a “high-viral-load individual” as a subject with plasma HIV-1 RNA levels ≥50,000 (4.7 log_10_) copies/ml at a given test time-point. We defined the “period of high transmissibility,” or “duration of high viral load,” as the time period during which an HIV-infected individual has plasma HIV-1 RNA ≥50,000 (4.7 log_10_) copies/ml. For the 14 seroconverters with high early HIV-1 RNA levels in the Tshedimoso study, we estimated the duration of high viral load in the absence of cART using cubic smoothing splines for those with more than 5 data points, and ordinary least squares regression for those with fewer data points. For individuals with increasing HIV-1 RNA levels, the duration of high viral load was imputed as the time from seroconversion to the last observation prior to cART initiation. In the sensitivity analysis, the duration of high viral load for subjects with increasing HIV RNA (n = 3) was estimated using the Kaplan-Meier method. To describe the procedure for estimating the potential reduction in the period of high viral load, we introduce some notation: Let X denote the time when new infections occur and Y denote the duration of high viral load. We assume that X follows a uniform distribution within the testing interval and is independent of Y. The distribution of Y is estimated from the empirical distribution based on the 14 seroconverters. Using τ to denote the length of testing interval, the proportion of individuals with high viral load who can be identified by using repeated HIV testing at τ-month interval is Pr(X+Y> τ), the probability of X+Y being greater than τ. The potential reduction in the period of high HIV transmissibility in individuals with high viral load that can be achieved by repeated HIV testing and ARV treatment was approximated by E(X+Y-τ|X+Y-τ ≥0), the expected value of X+Y-τ when it is positive. Confidence intervals for these two quantities were derived using the bootstrap method [46]. All reported p-values are 2-sided and not adjusted for multiple comparisons.

## Results

Baseline HIV-1 RNA levels were quantified in 4,348 drug-naïve HIV-infected individuals who participated in seven clinical research studies in Botswana. Two (Mashi and Mma Bana) were MTCT cohorts; two (Botsogo and Dikotlana) were general population cohorts comprising asymptomatic HIV-positive individuals; and three (Tshepo, Mashi+, and Bomolemo) were cART-initiating cohorts. Although time of HIV infection for participants within these cohorts was unknown, the CD4-based inclusion criteria were used at enrollment. Therefore, it is likely that the times from infection are shorter for subjects in the general and MTCT cohorts than for those in the cART-initiating cohort, as illustrated in the supplementary Figure S2.

**Figure 1:**
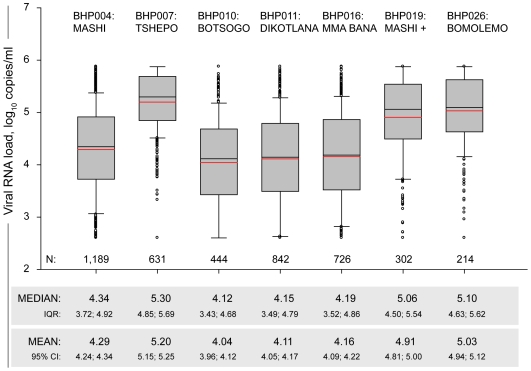
HIV-1 subtype C RNA levels in seven BHP cohorts (pre-cART baseline data). In the box plots, the boundary of the box closest to zero indicates the 25^th^ percentile, a black line within the box marks the median, a red line within the box marks the mean, and the boundary of the box farthest from zero indicates the 75^th^ percentile. Whiskers above and below the box indicate the 10^th^ and 90^th^ percentiles. Points above and below the whiskers indicate outliers outside the 10^th^ and 90^th^ percentiles. Text above each box plot indicates the BHP study number and the name of the corresponding cohort. Numbers of included participants per cohort are shown at the bottom within the graph. Median, IQR, mean, and 95% CI are presented at the bottom outside the graph.

The baseline levels of HIV-1 RNA in seven BHP cohorts are presented in Figure 1. Both median and mean values ranged within about one log_10_ copies/ml among analyzed BHP cohorts, from 4.12 log_10_ in the Botsogo cohort to 5.30 log_10_ in the Tshepo cohort. The lowest values were in the general population cohorts, Botsogo and Dikotlana, with medians (IQR) of 4.12 (3.43; 4.68) log_10_ and 4.15 (3.49; 4.79) log_10_, respectively. The MTCT cohorts were close to the general population cohorts with slightly elevated median and mean values, although the differences were statistically significant between Mashi and Botsogo (p<0.001), between Mashi and Dikotlana (p<0.001), and between Mma Bana and Botsogo (p = 0.026); the difference between Mma Bana and Dikotlana was not significant. As expected, the levels of HIV-1 RNA were significantly higher in the cART-initiating cohorts, Tshepo, Mashi+, and Bomolemo (all p-values between any cART-initiating cohort and any general population or MTCT cohort were less than 0.00001).

**Figure 2:**
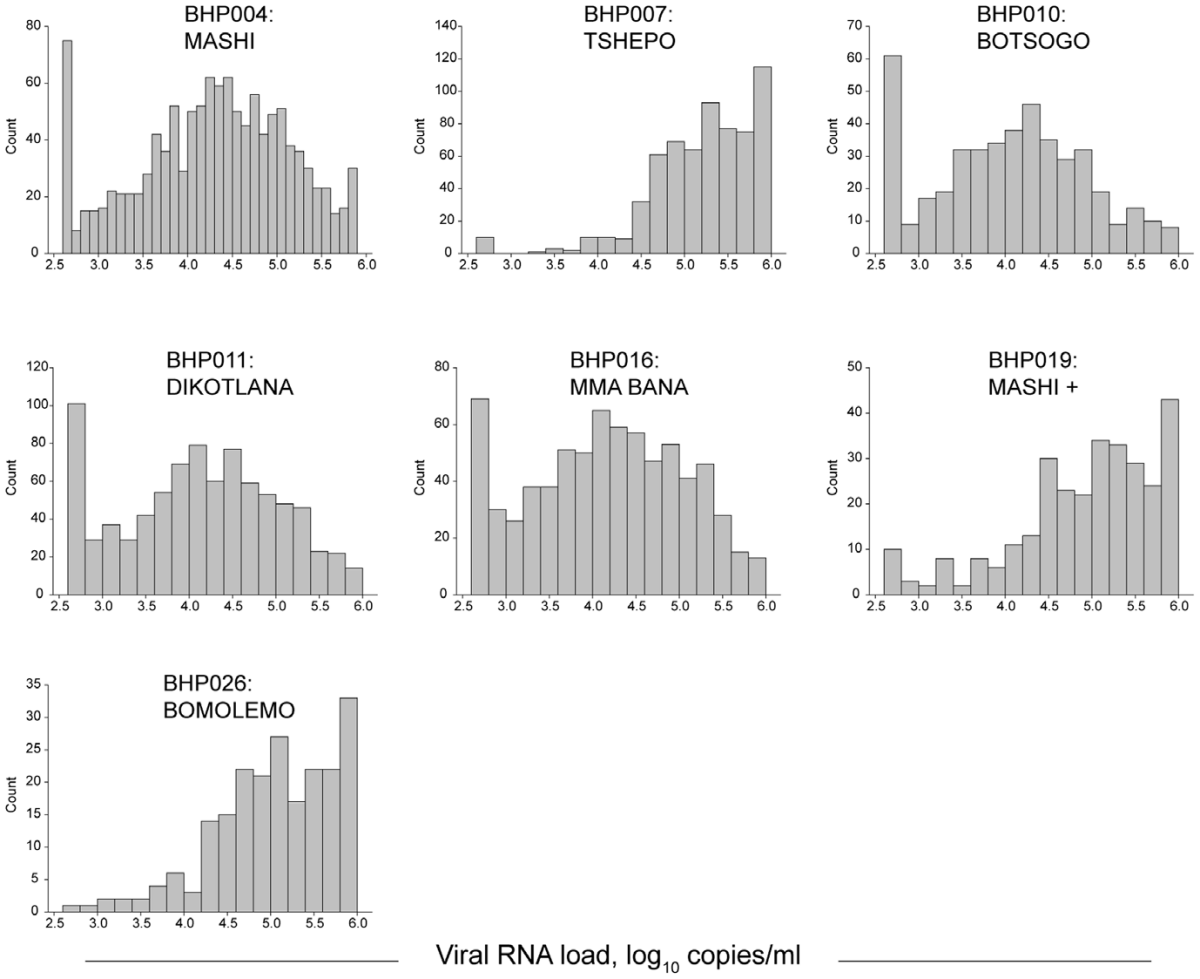
Distribution of HIV-1 RNA levels in seven BHP cohorts (pre-cART baseline data). Y-axis shows the count, and differs between cohorts. X-axis denotes log_10_ HIV-1 RNA levels; the scale is uniform for all cohorts. Labels in each graph indicate the BHP study number and the name of the corresponding cohort.

The distribution of plasma HIV-1 RNA among BHP cohorts is shown in Figure 2. Deviation from a normal distribution was evident for each cohort, and the observed patterns were common within the categories of general population cohorts, MTCT cohorts, and cART-initiating cohorts. The HIV-1 RNA distribution comprising the Botsogo, Dikotlana, Mashi, and Mma Bana cohorts were close to the normal “bell-like” shape of distribution, but were enriched by HIV-infected individuals with low/undetectable levels of viral load, which was evident from spikes at the left side of the histograms representing these cohorts. In contrast, the three cART-initiating cohorts, Tshepo, Mashi+, and Bomolemo, demonstrated deviation from a normal distribution of plasma HIV-1 RNA and were skewed to the right part of the histograms, providing evidence that these cohorts were over-represented by HIV-infected individuals with high viral loads. The normality test failed for all cohorts (p = 0.0027 for Mma Bana, and p<0.001 for all other cohorts), suggesting that the HIV RNA load in the BHP cohorts analyzed were not normally distributed. The observed lack of normal distribution of plasma HIV-1 RNA can be explained, at least in part, by varying inclusion criteria for each of the different cohorts. To address this, the baseline CD4+ cell counts data for each cohort are presented in Supplementary Table S2. Due to the known inverse correlation between CD4+ cell counts and plasma HIV-1 RNA levels, the specified levels of CD4+ cell counts at enrollment are likely to contribute to the observed lack of normal distribution of HIV-1 RNA levels. In addition, spikes at the edges of histograms can be explained by censoring of the data at low and high thresholds of HIV-1 RNA quantification.

We analyzed the proportion of HIV-infected individuals within each cohort with a pre-cART HIV-1 RNA level exceeding three thresholds: ≥10,000 (4.0 log_10_) copies, ≥50,000 (4.7 log_10_) copies, and ≥100,000 (5.0 log_10_) copies (Table 1). Consistent with the analysis of levels and distribution, the proportion of individuals exceeding each threshold was lowest in the general population cohorts, Botsogo and Dikotlana, followed by the MTCT cohorts, Mashi and Mma Bana, and was highest among the cART-initiating cohorts, Tshepo, Mashi+, and Bomolemo. The proportion of individuals with HIV-1 RNA ≥50,000 (4.7 log_10_) copies ranged from 24%–28% in the general population cohorts to 65%–83% in the cART-initiating cohorts.

**Table 1:** Proportion of HIV-infected individuals with high levels of HIV-1 RNA in seven BHP cohorts.

	BHP cohorts						
HIV-1 RNA, copies/ml	Mashi	Tshepo	Botsogo	Dikotlana	Mma Bana	Mashi +	Bomolemo
≥10,000	66%	96%	54%	57%	58%	87%	92%
≥50,000	34%	83%	24%	28%	30%	65%	72%
≥100,000	22%	67%	14%	18%	20%	54%	57%

**Figure 3:**
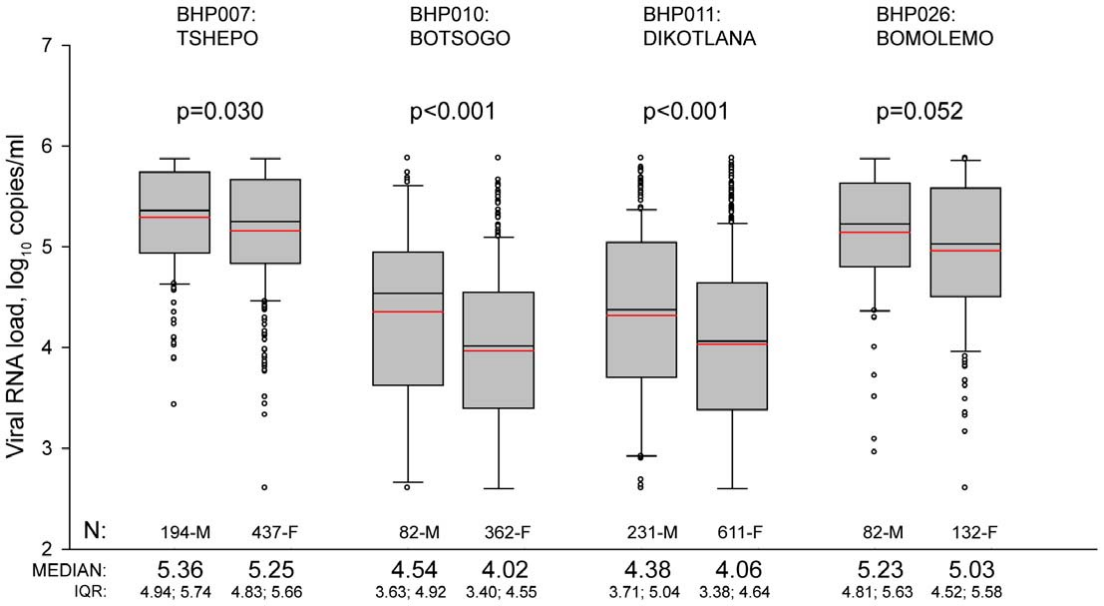
Gender differences in HIV-1 RNA levels in four BHP cohorts (pre-cART baseline data). For explanation of box plots see Figure 1 legend. Text above each box plot indicates the BHP study number and the name of the corresponding cohort. Comparison of HIV-1 RNA levels between genders was performed by the Mann-Whitney Rank Sum test, and p-values are presented above the box plots. Numbers of included male (M) and female (F) participants per cohort are shown at the bottom within the graph. Median and IQR are presented at the bottom outside the graph.

Potential gender differences in levels of HIV-1 RNA were analyzed in four cohorts: Tshepo, Botsogo, Dikotlana, and Bomolemo (the three remaining cohorts comprised only females). The results of HIV-1 RNA levels comparisons between genders are presented in Figure 3. Male participants had a higher HIV-1 RNA levels in plasma than female participants. In the general population cohorts, Botsogo and Dikotlana, there was a significant difference of about 0.3–0.5 log_10_ between genders (p< 0.001), while in the two cART-initiating cohorts, Tshepo and Bomolemo, we observed smaller differences of about 0.1–0.2 log_10_ (p = 0.030 and p = 0.052 for Tshepo and Bomolemo cohorts, respectively). Analysis of CD4+ cell values revealed no statistically significant gender difference in three out of four cohorts (data not shown). In the fourth cohort, Bomolemo, male participants had lower values of CD4+ cells than female participants (p = 0.001).

No associations were found between HIV-1 RNA levels and age in five of the seven analyzed cohorts (data not shown). A weak direct association was found in the Botsogo and Dikotlana cohorts (r = 0.099, p = 0.038, and r = 0.072, p = 0.036, respectively). A weak to moderate inverse association was found between HIV-1 RNA levels and CD4+ cell counts that was statistically significant in all analyzed cohorts (Supplementary Table S3; p = 0.030 in Mashi+, and p<0.001 for all other cohorts). For every 1.0 log_10_ increase in HIV-1 RNA, the loss in CD4+ cells ranging from 21.1 to 98.8 CD4+ cells (Supplementary Table S3).

**Figure 4:**
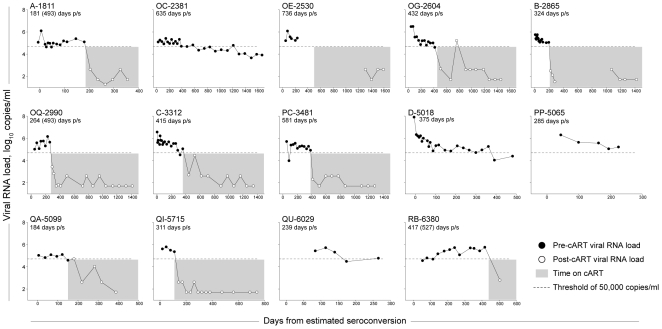
Dynamics of HIV-1 RNA levels in subjects with high early viral set point, n = 14. High HIV RNA level was considered at ≥50,000 (4.7 log_10_) copies/ml. Early viral set point was determined as a mean value from 50 to 200 days p/s. Data for each subject is presented in a separate graph. Patient code is shown at the top left of each graph. Y-axis shows HIV-1 RNA levels, log_10_ copies/ml; scale is uniform for all subjects; the 50,000 (4.7 log_10_) copy threshold is shown as a dashed line. X-axis denotes days from estimated seroconversion; scale differs between subjects due to differences in the follow-up period. Filled circles delineate pre-cART HIV-1 RNA values, and open circles show post-cART HIV-1 RNA values. Time of cART is highlighted by gray zone, if applicable. For calculation of slopes and prediction of time of decline to the threshold of 50,000 (4.7 log_10_) copies, only pre-cART data were used. The conservative estimate of individual time to decline below 50,000 (4.7 log_10_) copies is shown under the patient code with the less conservative Kaplan-Meier estimate in brackets (if it differs from the conservative estimate).

To assess the duration of high HIV-1 RNA levels following initial infection with HIV-1 subtype C, a primary infection cohort of subjects enrolled before or within a short time after infection [27], [39], [43], [44], [45] was utilized (Tshedimoso study). Early viral set point was defined as mean viral RNA from 50 to 200 days post-seroconversion (p/s) [27], [39], and was ≥50,000 (4.7 log_10_) copies/ml in 14 of 42 (33%; 95% CI: 20%–50%) subjects. The observed dynamics of HIV-1 RNA levels in the subset of 14 subjects are presented in Figure 4. Ten of the 14 subjects initiated cART once their CD4+ cell counts fell below the threshold level indicating treatment. The pre-cART HIV-1 RNA data were used to estimate the duration of high viral load in the absence of cART. In three cases, subjects A-1811, OQ-2990, and RB-6380, the pre-cART HIV-1 RNA slopes had positive values, and their duration of high viral load were estimated to be the time interval to the last observation prior to initiation of cART. The mean (95% CI) and median (IQR) for duration of high viral load were 384 (296; 472) days p/s, and 350 (269; 428) days p/s.

**Figure 5:**
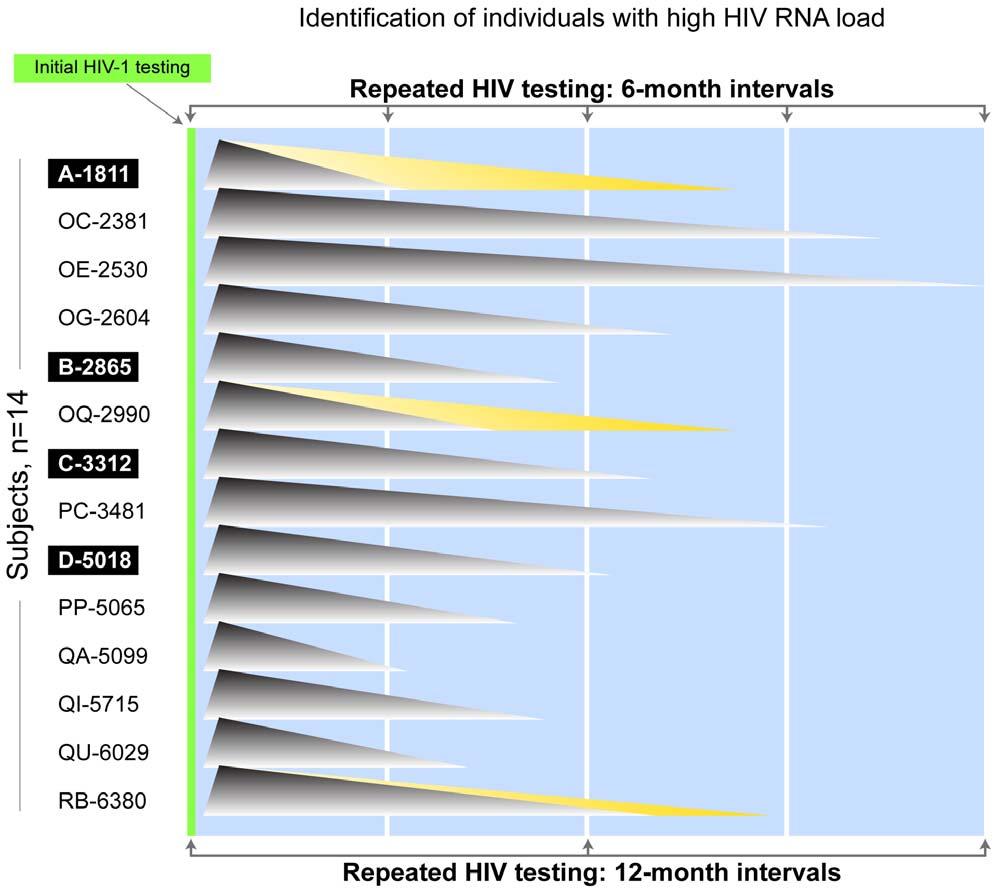
Identification of high HIV-1 RNA individuals by repeated HIV testing: six-month intervals vs. twelve-month intervals. All subjects are assumed to be HIV-seronegative at initial HIV testing, and to acquire HIV-1 infection shortly after that. The study subjects' code is shown at the left of the graph, and four acutely infected individuals are highlighted. The high viral load for each subject is presented as a shaded triangle symbolizing the “tip of the viral load iceberg”. The base of each triangle corresponds to the estimated time of dropping HIV-1 RNA levels below 50,000 (4.7 log_10_) copies/ml for each subject. In all subjects estimation of high viral load duration is outlined by gray shading delineating the time from seroconversion to the last observation before cART. In addition, in three subjects —A-1811, OQ-2990, and RB-6380—yellow shading corresponds to estimation of high viral load duration using the Kaplan-Meier method. Six-month interval HIV testing is delineated at the top, and 12-month interval testing is shown at the bottom.

Assuming that HIV-infected individuals with high viral load may contribute disproportionally to HIV transmission, two questions related to public health interventions were addressed. To assess the proportion of individuals wih high HIV-1 RNA levelsthat can be identified at selected intervals (with intent to initiate cART), we tested 6- and 12-month-interval algorithms of repeated HIV testing in the community (Fig. 5), under the assumption that the empirical distribution of the durations of high viral load for these 14 subjects is a good approximation to the true distribution. Because every high viral load subject was observed or predicted to remain above 50,000 (4.7 log_10_) copies/ml for 6 months or more, HIV testing every 6 months would be able to identify all of them. In the case of 12-month-interval testing, 85% (95% CI: 77%–94%) of high viral load individuals can be identified. We used the same 6- and 12-month-interval HIV testing algorithms to estimate the fraction of the period of high transmissibility that would be eliminated by immediately treating identified high viral load individuals (with intent to initiate cART) in the community. It was estimated that 77% (95% CI: 71%–82%) of the period of high transmissibility could be eliminated by 6-month-interval testing and 56% (95% CI: 47%–64%) by 12-month-interval testing.

We note that for those subjects with increasing HIV-1 RNA over time, using time to the last observation prior to initiation of cART as their high viral load duration led to conservative estimates of the fraction of identified subjects. This approach also underestimates the fraction of higher-risk transmission time that can potentially be eliminated by initiation of cART. In a sensitivity analysis using a less conservative Kaplan-Meier approach, we estimated that the high viral load durations of these individuals changed from 181 to 493 days, 264 to 493 days, and 417 days to 527 days, respectively (Fig. 5). The high viral load durations for the remaining 11 subjects remain unchanged. Based on this set of high viral load durations, we obtain similar results as before. More specifically, the fraction of HIV-infected individuals with high viral load that can be identified by repeated 6-month-interval testing remained at 100%, and the fraction of potential high-risk HIV transmission time that can be reduced was 79% (95% CI: 75%–83%). The fraction of individuals with high viral load that can be identified by 12-month-interval testing was 91% (95% CI: 83%–99%), and the fraction of potential high-risk HIV transmission time that can be eliminated was estimated at 59% (95% CI: 53%–66%).

## Discussion

The Botswana population is one of those most severely affected by HIV-1 subtype C infection. To assess the levels and distribution of HIV-1 subtype C RNA in plasma, the analysis was performed utilizing existing data from three types of cohorts: general population, MTCT, and cART-initiating cohorts. Because of their size and breadth, these cohorts adequately represent the entire population in the local HIV/AIDS epidemic. The HIV-1 RNA data was presented per cohort to highlight the heterogeneity of HIV-1 RNA levels *between* different cohort types in contrast to the relative homogeneity *within* each cohort type. We found a one log_10_ difference of HIV-1 RNA levels between, and a differential distribution of HIV-1 RNA levels within, different types of cohorts. The variation in HIV-1 RNA levels between cohorts observed in this study suggests that one should use caution when comparing different types of cohorts of HIV-infected individuals, even those originating from the same geographic area and infected with the same HIV-1 subtype.

Our analysis provides evidence that a substantial proportion of HIV-1 subtype C-infected individuals have high HIV-1 RNA levels. Although time of infection was not known in seven analyzed cohorts, it is likely that some individuals with high HIV-1 RNA levels had been infected for a long time before enrolling in the research studies. Given the fact that approximately 25% of subjects in the general population cohorts have HIV-1 RNA levels above 50,000 (4.7 log_10_) copies/ml, there is a possibility that in a majority of the 33% of seroconverters who had high early HIV-1 RNA levels, the viral load would never drop below 50,000 (4.7 log_10_) copies/ml without extensive monitoring (as per study protocol) and initiation of ARV treatment. The high proportion of individuals with elevated levels of HIV-1 RNA deserves further attention and design of interventions targeting individuals with high viral load.

In the cohort of individuals acutely or recently infected by HIV-1 subtype C, we observed that 33% (95% CI: 20%–50%) of individuals maintained HIV-1 RNA levels of ≥50,000 (4.7 log_10_) copies/ml. Identifying HIV-infected individuals who maintain high levels of viral load for an extended period of time and intervening among them, including treating them with ARVs along with behavioral modification, might be an important public health HIV prevention strategy because such individuals are likely to transmit HIV more efficiently than those who maintain viremia at lower levels [4], [5]. This fraction of HIV-infected individuals with elevated levels of viral RNA for extended periods of time may be responsible for a high proportion of HIV transmissions in the community. If the hypothesis that individuals with high HIV-1 RNA levels are fueling HIV epidemic is true, the strategy for identifying HIV-infected individuals with high viral loads followed by initiation of cART might represent a modified and more practical version of the “test-and-treat” approach [6].

Longitudinal data from the cohort of acutely and recently infected individuals allowed us to estimate the duration of the time with viral loads remaining above 50,000 (4.7 log_10_) copies/ml, the proportion of individuals with high viral loads that can be identified using repeated HIV testing, and the potential reduction of the period of high HIV transmissibility that can be achieved by repeated HIV testing and treating in the community. The mean durations of approximately 384 days p/s and median of 350 days p/s are conservative estimates for time for maintaining viral RNA ≥50,000 (4.7 log_10_) copies/ml because for those whose viral loads had an increasing trend before starting cART, the duration of high viral load was taken to be time from seroconversion to last observation prior to cART. This interval provides a lower bound for the true duration. Our analysis suggests that repeated HIV testing in the community could identify a high proportion of infected individuals with high viral loads if the interval between HIV tests is approximately 6 months. This approach could also reduce the period during which individuals with high HIV-1 RNA levels can transmit virus with immediate cART initiation follows HIV testing.

We observed higher HIV-1 RNA levels in men in two cohorts representing the general population and low or no gender difference in the two cART-initiating cohorts. Gender differences in the levels of HIV-1 RNA were described previously [47], [48], [49]. Despite the initial levels of HIV-1 RNA being lower in women than in men, the rates of progression to AIDS did not differ [47]. The gender differences in viral load might have implications for initiation of cART, if the treatment strategy is based on the levels of HIV-1 RNA. Conversely, a selection bias in different cohorts cannot be completely excluded. In future studies, it would be important to determine whether the rate of HIV transmission differs between genders with similar levels of HIV-1 RNA.

The limitations of the current study include the small sample size in the primary infection cohort and unknown time of HIV infection in the large cohorts representing later time points over the course of infection. The relatively small sample size (n = 42) of the primary infection cohort reflects well known challenges in identifying acutely infected individuals which include but are not limited to a lack of specific clinical signs and symptoms, and the extremely short time period preceding seroconversion. To reflect the uncertainty of the analyses associated with relatively small sample, we included 95% confidence intervals and/or inter-quartile ranges for all analyses throughout the paper. Another limitation of the study is unknown time of infection in the large cohorts where baseline pre-cART data was used for analysis. The analyzed data represent snapshots of HIV-1 RNA levels at different time points in the HIV/AIDS epidemic in Botswana spanning the time period from 2000 to 2009. The concern of unknown time from infection could be lessened, at least partially, by grouping cohorts (as presented in Fig. S2), which was largely driven by the CD4-based inclusion criteria. Conversely, the large amount of information presented on HIV-1 subtype C RNA levels from existing carefully monitored studies that target different subsets of population in one geographic region infected with a single HIV-1 subtype is a strength of the analysis performed.

Although the cost-effectiveness was outside the scope of the current study, the ultimate goal of our research is to develop cost-effective means for mitigation or control of HIV infection in the community. Early treatment for HIV can be cost-effective by virtue of greatly reducing the need for treatment of opportunistic infections and decreasing mortality. In fact, the per-person survival gains with cART greatly exceed many other therapeutic approaches [50]. Mathematical modeling supports early initiation of cART, genotypic testing in treatment-experienced and treatment-naïve patients, and expanded programs for HIV screening and linkage for care [50] as appropriate cost-effective public health approaches for better control of the HIV/AIDS epidemic.

When data are available on transmission incidence as a function of HIV-1 RNA levels and other factors in Botswana, the analysis can be extended to directly estimate the numbers of transmissions per month averted by testing-and-treating of individuals with high HIV-1 RNA levels under different testing schedules. In the meantime, under the assumptions that no high HIV-1 RNA individuals transmit once placed on cART and that transmission incidence is constant during the period of high viral load, the quantity that we were able to estimate (the fractionate reduction in the period of high HIV-1 RNA levels due to test-and-treat) usefully measures the fractionate reduction in transmission incidence during the period of high HIV-1 RNA levels.

In summary, we suggest that HIV testing aimed at identifying and offering cART to HIV-infected individuals with high viral load could be a reasonable goal in the global fight to reduce HIV incidence. If HIV-infected individuals maintaining high levels of HIV-1 RNA for extended period of time contribute disproportionally to HIV transmission, a modified “test-and-treat” strategy targeting such individuals by repeated HIV testing (followed by initiation of cART) might be a useful public health strategy for mitigating the HIV epidemic in some communities, particularly those with high HIV prevalence. A small sample size in this study is a limitation of the estimated parameters of interest. It would be important to apply similar analyses to other existing larger sample sets in the HIV-1 subtype B (e.g., VanGen efficacy trials) and non-subtype B (e.g., CHAVI cohorts) settings.

## Supporting Information

Figure S1 Time points of sampling and HIV-1 RNA testing in the primary infection cohort (n = 42). The time scale is set to the estimated time of seroconversion as time 0. The sampling time points were limited to 500 days p/s. The study subjects' code is shown in the column at the left. Eight acutely infected subjects are highlighted. Fourteen HIV-infected individuals with HIV-1 RNA levels ≥50,000 (4.7 log_10_) copies/ml are shown with arrows preceding the subjects' code. Gray bars indicate time on cART in ten subjects (delineated by arrows on the right).
(9.90 MB TIF)

Figure S2 Different types of cohorts over the course of HIV-1 infection: a simplistic scheme illustrating relative distribution of the time from HIV infection. Time of HIV infection was estimated for all participants in the primary HIV-1 infection cohort, and the follow-up period is shown by gray rectangle. Time of HIV infection was unknown for other cohorts. The distribution of the time from HIV infection is outlined for the general population cohorts by green curve, for the MTCT cohorts by blue curve, and for the cART-initiating cohorts by black curve. Red lines delineate the dynamics of plasma HIV-1 RNA levels over the course of infection. Three HIV-1 RNA curves represent high, medium and low viral set points over primary HIV infection, chronic HIV infection, and progression to AIDS stages, respectively.
(10.04 MB TIF)

Table S1 Time of enrollment.
(0.03 MB DOC)

Table S2 Baseline (per-ART) CD4+ cell counts.
(0.03 MB DOC)

Table S3 Slopes and analysis of potential associations (Spearman rank test) between HIV-1 subtype C RNA levels and baseline CD4+ cell count in seven BHP cohorts.
(0.03 MB DOC)
