# Supplementary figures and images for: HIV-1 Subtype C-Infected Individuals Maintaining High Viral Load as Potential Targets for the “Test-and-Treat” Approach to Reduce HIV Transmission
Source: PLoS One. 2010 Apr 12;5(4):e10148. doi: 10.1371/journal.pone.0010148 (PMC2853582; doi:10.1371/journal.pone.0010148)

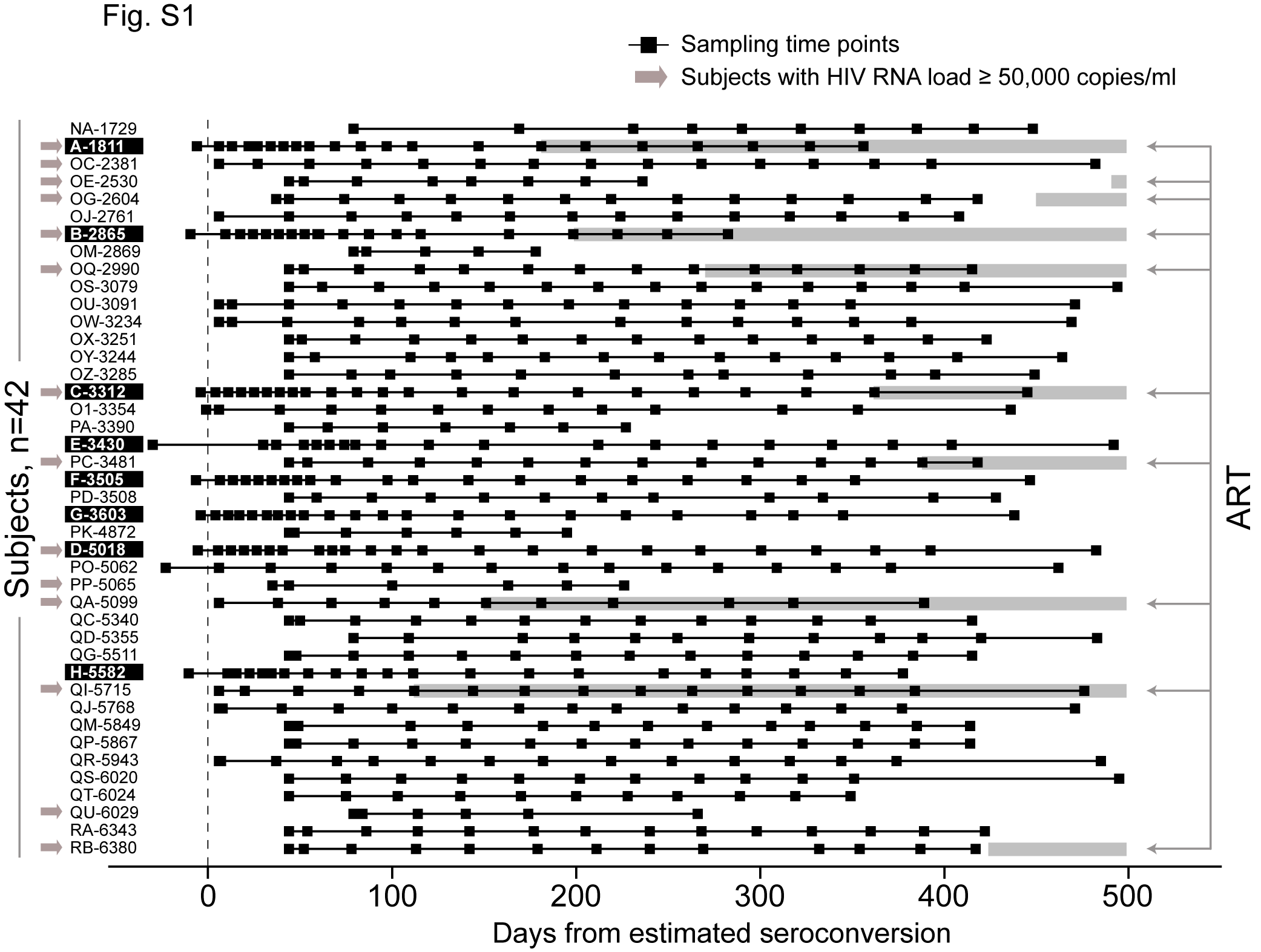

Supplement: Figure S1 — Time points of sampling and HIV-1 RNA testing in the primary infection cohort (n = 42). The time scale is set to the estimated time of seroconversion as time 0. The sampling time points were limited to 500 days p/s. The study subjects' code is shown in the column at the left. Eight acutely infected subjects are highlighted. Fourteen HIV-infected individuals with HIV-1 RNA levels ≥50,000 (4.7 log10) copies/ml are shown with arrows preceding the subjects' code. Gray bars indicate time on cART in ten subjects (delineated by arrows on the right). (9.90 MB TIF) [file pone.0010148.s001.tif]

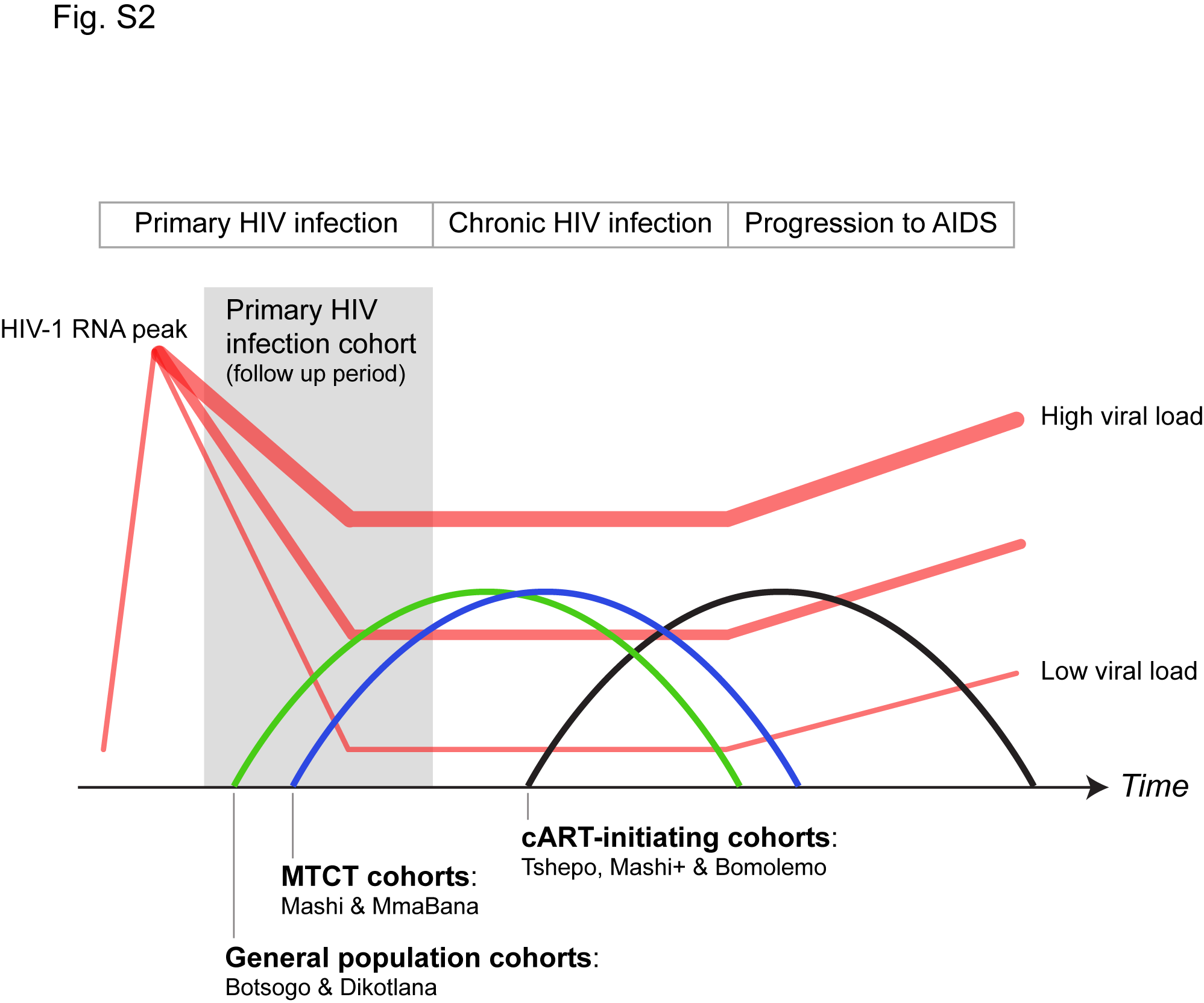

Supplement: Figure S2 — Different types of cohorts over the course of HIV-1 infection: a simplistic scheme illustrating relative distribution of the time from HIV infection. Time of HIV infection was estimated for all participants in the primary HIV-1 infection cohort, and the follow-up period is shown by gray rectangle. Time of HIV infection was unknown for other cohorts. The distribution of the time from HIV infection is outlined for the general population cohorts by green curve, for the MTCT cohorts by blue curve, and for the cART-initiating cohorts by black curve. Red lines delineate the dynamics of plasma HIV-1 RNA levels over the course of infection. Three HIV-1 RNA curves represent high, medium and low viral set points over primary HIV infection, chronic HIV infection, and progression to AIDS stages, respectively. (10.04 MB TIF) [file pone.0010148.s002.tif]
