# Supplementary material for: HIV-1 Subtype C-Infected Individuals Maintaining High Viral Load as Potential Targets for the “Test-and-Treat” Approach to Reduce HIV Transmission
Source: PLoS One. 2010 Apr 12;5(4):e10148. doi: 10.1371/journal.pone.0010148 (PMC2853582; doi:10.1371/journal.pone.0010148)
Supplement: Table S1 — Time of enrollment. (0.03 MB DOC) [file pone.0010148.s003.doc]

**Table S1. Time of enrollment.**

| **Cohort** | **Start** | **End** |
| --- | --- | --- |
| BHP004 Mashi | March 2000 | June 2003 |
| BHP007 Tshepo | December 2002 | December 2004 |
| BHP010 Dikotlana | April 2005 | January 2006 |
| BHP011 Botsogo | January 2005 | July 2007 |
| BHP016 Mma Bana | May 2006 | April 2008 |
| BHP019 Mashi+ | February 2002 | October 2007 |
| BHP026 Bomolemo | November 2008 | April 2009 |
