# Supplementary material for: HIV-1 Subtype C-Infected Individuals Maintaining High Viral Load as Potential Targets for the “Test-and-Treat” Approach to Reduce HIV Transmission
Source: PLoS One. 2010 Apr 12;5(4):e10148. doi: 10.1371/journal.pone.0010148 (PMC2853582; doi:10.1371/journal.pone.0010148)
Supplement: Table S2 — Baseline (per-ART) CD4+ cell counts. (0.03 MB DOC) [file pone.0010148.s004.doc]

**Table S2. Baseline (per-ART) CD4+ cell counts.**

| **Cohort** | **Mean** | **95% CI** | | **Median** | **25^th^ percentile** | **75^th^ percentile** |
| --- | --- | --- | --- | --- | --- | --- |
| BHP004 Mashi | 397 | 384; | 410 | 367 | 235 | 515 |
| BHP007 Tshepo | 218 | 205; | 231 | 208 | 142 | 283 |
| BHP010 Dikotlana | 514 | 492; | 536 | 468 | 377 | 598 |
| BHP011 Botsogo | 461 | 449; | 473 | 420 | 334 | 549 |
| BHP016 Mma Bana | 364 | 350; | 378 | 340 | 213 | 482 |
| BHP019 Mashi+ | 336 | 293; | 379 | 222 | 154 | 336 |
| BHP026 Bomolemo | 151 | 138; | 164 | 154 | 70 | 215 |
