# Supplementary material for: HIV-1 Subtype C-Infected Individuals Maintaining High Viral Load as Potential Targets for the “Test-and-Treat” Approach to Reduce HIV Transmission
Source: PLoS One. 2010 Apr 12;5(4):e10148. doi: 10.1371/journal.pone.0010148 (PMC2853582; doi:10.1371/journal.pone.0010148)
Supplement: Table S3 — Slopes and analysis of potential associations (Spearman rank test) between HIV-1 subtype C RNA levels and baseline CD4+ cell count in seven BHP cohorts. (0.03 MB DOC) [file pone.0010148.s005.doc]

**Table S3. Slopes and analysis of potential associations (Spearman rank test)** between HIV-1 subtype C RNA levels and baseline CD4+ cell count in seven BHP cohorts.

|  | **BHP cohorts** | | | | | | |
| --- | --- | --- | --- | --- | --- | --- | --- |
| **Parameter** | **Mashi** | **Tshepo** | **Botsogo** | **Dikotlana** | **Mma Bana** | **Mashi +** | **Bomolemo** |
| Slopes, CD4+ cells per 1.0 log_10_ HIV RNA | -98.8 | -46.5 | -47.1 | -79.4 | -88.7 | -21.1 | -53.4 |
| Correlation coefficient | -0.390 | -0.280 | -0.316 | -0.381 | -0.410 | -0.165 | -0.431 |
| p-value | <0.001 | <0.001 | <0.001 | <0.001 | <0.001 | 0.030 | <0.001 |
